# Chiropractic maintenance care - what’s new? A systematic review of the literature

**DOI:** 10.1186/s12998-019-0283-6

**Published:** 2019-11-21

**Authors:** Axén Iben, Hestbaek Lise, Leboeuf-Yde Charlotte

**Affiliations:** 1 Karolinska Institutet, Institute of Environmental Medicine, Unit of Intervention and Implementation Research for Worker Health, Nobels väg 13, 171 77 Stockholm, Sweden; 2 Et Liv I Bevegelse (The Norwegian Chiropractic Research Foundation), Storgt 10a, 0155 Oslo, Norway; 3 Deptartment of Sport Science and Clinical Biomechanics, Odense, Denmark; 4 Nordic Institute of Chiropractic and Clinical Biomechanics, Odense, Denmark; 5 Institute for Regional Health Research, University of Southern Denmark, Odense, Denmark

**Keywords:** Maintenance care, Prevention, Chiropractic, Pain, Disease management, Systematic review

## Abstract

**Background:**

Maintenance Care is a traditional chiropractic approach, whereby patients continue treatment after optimum benefit is reached. A review conducted in 1996 concluded that evidence behind this therapeutic strategy was lacking, and a second review from 2008 reached the same conclusion. Since then, a systematic research program in the Nordic countries was undertaken to uncover the definition, indications, prevalence of use and beliefs regarding Maintenance Care to make it possible to investigate its clinical usefulness and cost-effectiveness. As a result, an evidence-based clinical study could be performed. It was therefore timely to review the evidence.

**Method:**

Using the search terms “chiropractic OR manual therapy” AND “Maintenance Care OR prevention”, PubMed and Web of Science were searched, and the titles and abstracts reviewed for eligibility, starting from 2007. In addition, a search for “The Nordic Maintenance Care Program” was conducted. Because of the diversity of topics and study designs, a systematic review with narrative reporting was undertaken.

**Results:**

Fourteen original research articles were included in the review. Maintenance Care was defined as a secondary/tertiary preventive approach, recommended to patients with previous pain episodes, who respond well to chiropractic care. Maintenance Care is applied to approximately 30% of Scandinavian chiropractic patients. Both chiropractors and patients believe in the efficacy of Maintenance Care. Four studies investigating the effect of chiropractic Maintenance Care were identified, with disparate results on pain and disability of neck and back pain. However, only one of these studies utilized all the existing evidence when selecting study subjects and found that Maintenance Care patients experienced fewer days with low back pain compared to patients invited to contact their chiropractor ‘when needed’. No studies were found on the cost-effectiveness of Maintenance Care.

**Conclusion:**

Knowledge of chiropractic Maintenance Care has advanced. There is reasonable consensus among chiropractors on what Maintenance Care is, how it should be used, and its indications. Presently, Maintenance Care can be considered an evidence-based method to perform secondary or tertiary prevention in patients with previous episodes of low back pain, who report a good outcome from the initial treatments. However, these results should not be interpreted as an indication for Maintenance Care on all patients, who receive chiropractic treatment.

## Background

New evidence regarding the natural course of spinal pain should lead to a shift in treatment approaches. Previously, low back pain (LBP) and neck pain (NP) were thought to be self-limiting ailments, hardly worthy of attention. Consequently, treatment, if at all required, was aimed at shortening the course of symptoms. However, gradually the fact that spinal pain is a recurring disorder, as stated by van Korff more than 20 years ago [1], is gaining accept. The acute episode of spinal pain, similarly to an episode of asthma, may be short-lived, but the condition is often, as for asthma, life-long. With this new understanding of spinal pain as a condition with exacerbations and remissions throughout life [2], it might be wise to shift the focus of treatment from cure of the condition to management of pain trajectories [3].

Chiropractors appear to have been in the forefront in this domain. ‘Maintenance Care’ is a well-known concept in the chiropractic profession, describing continued care beyond that of reducing symptoms. However, it has been used in different ways. Some chiropractors appear to have recommended Maintenance Care as a form of precaution to keep the patient healthy, regardless of symptoms and patient history. Others though, seem to have used it to ‘keep patients going’, when they had chronic or recurring problems.

The former approach has been criticized for lack of evidence and considered mainly a financial model to keep the practice busy. The second approach, although sensible in the light of spinal pain being a recurrent and persistent condition, did not have any scientific support and was also often frowned upon.

Interestingly, although the term ‘Maintenance Care’ has been used for many decades and by chiropractors all over the world, there seemed to be no official definition nor any knowledge regarding its clinical usefulness. A narrative review from 1996 [4] concluded that: “there is no scientific evidence to support the claim that Maintenance Care improves health status”. A second review, 12 years later, did not find that there was much more knowledge available on this topic [5]. The conclusions were as follows:

- There was no evidence-based definition of Maintenance Care and the indications for and nature of its use remained to be clearly stated.
- Many chiropractors seemed to believe in the usefulness of Maintenance Care but there was little evidence how this was accepted by their patients.
- The prevalence with which Maintenance Care was used had not been established.
- Efficacy and cost-effectiveness of Maintenance Care for various types of conditions were unknown.

Further, both reviews recommended that the topic should be studied further to obtain better information. Specifically, a clinical trial based on evidence about indications and treatment approach was needed. Based on these recommendations, a research program called “The Nordic Maintenance Care Program” was launched with the aim to increase the knowledge regarding Maintenance Care, i.e. the information needed prior to performing a clinical trial to test its efficacy. This research program explored Maintenance Care among chiropractors in Denmark, Sweden, Finland, and Norway.

Ten years have passed since the latest review, the clinical trial has been performed and results are published. It therefore seemed timely to monitor the evidence that has emerged and to describe the use and usefulness of Maintenance Care in chiropractic practice. We performed an additional systematic search of the literature to see what was new and, because the topics and study designs were varied, we concentrated on the outcomes of the different studies and narratively summarized the results instead of using evidence tables.

The objectives of the systematic review were the same as those in the most recent review, namely:

1. To define the concept of Maintenance Care and the indications for its use.
2. To describe chiropractors’ belief in Maintenance Care and patients’ acceptance of it.
3. To establish the prevalence with which chiropractors use Maintenance Care and possible characteristics of the chiropractors associated with its use.
4. To determine its efficacy and cost-effectiveness for various types of conditions.

## Method

The search from the previous review was repeated on November 5th, 2018, but this time including also the term “manual therapy”. Thus, the terms “chiropractic OR manual therapy” AND “Maintenance Care OR prevention” were entered both as MeSH-terms and free text. Only articles published after the search of the previous review, i.e. after 2007, were included. PubMed and Web of Science were searched. The search strategy is found in Additional file 1.

Only research studies were included, i.e. case studies, commentaries, and study protocols were excluded. Studies specifically referring to “wellness” were also excluded, as this term, in our opinion, refers to treatment aimed at improving health status before any symptoms arise (primary prevention), which has been found to be unsupported by evidence [6]. In other words, we wanted to concentrate this review on treatment related to symptoms.

In addition, a search was performed in PubMed using the term “The Nordic Maintenance Care Program”, as we knew that several relevant articles were published under that heading.

The first screening of titles and abstracts in PubMed was done independently by two authors (IA and CLY), and the search from Web of Science was done independently by another pair (IA and LH). Their findings were compared and agreed upon, if necessary, with the assistance of the third author.

The included studies were reviewed for their ability to answer the proposed research questions. This was done by all three authors independently, and the information obtained was compared and discussed, if necessary. The material was then summarized and reported narratively.

## Results

**Fig. 1:**
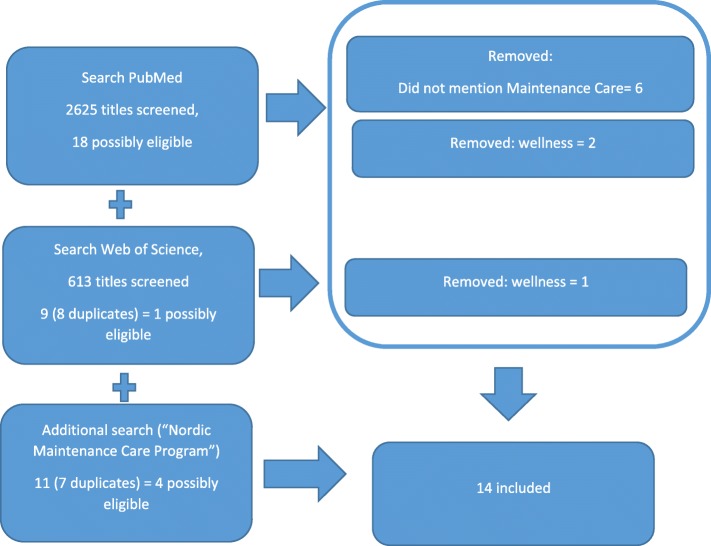
Search of databases, screening of titles, abstracts and articles, as well as reasons for excluding articles for a systematic review on chiropractic Maintenance Care

The PubMed and Web of Science resulted in ten potential papers and the Nordic Maintenance Care Program-search resulted in another four potential articles, i.e. 14 in total (please see Fig. 1).

The 14 included articles were published between 2008 and 2018. The majority (10/14) were from the Nordic Maintenance Care Program, specifically designed to address the knowledge gaps identified in the review from 2008. The remaining articles were from Canada, the US and Egypt. There was a mixture of qualitative (focus groups and interviews) and quantitative studies (surveys, observational studies and randomized controlled trials). One study was described as a structured workshop, but it was designed like a focus group discussion with a resulting qualitative summary [7].

Eight studies collected their data from chiropractors [8–15], who either estimated their responses or consulted their patient files, four studies collected their data from patients [16–19], in one study data were collected from both chiropractors and their patients [20], and one study used workers’ compensation claims data [21]. Please see Table 1 for a description of the included studies.

**Table 1:** Description of 14 studies published between 2008 and 2018, included in a systematic review on chiropractic Maintenance Care

Author Year of publication	Design of study	Population	Sample size	Response rate	Research question explored
Axén 2008 [8]	Survey	Chiropractors	*N* = 59	60%	Indications for Maintenance Care Prevalence of Maintenance Care
Axén 2009 [10]	Focus group + Survey	Chiropractors	*N* = 36 *N* = 129	22% 77%	Indications for Maintenance Care Chiropractors’ belief in Maintenance Care
Møller 2009 [11]	Survey with open-ended question	Chiropractors	*N* = 11	NA, selected group	Definition of concept Indications for Maintenance Care Prevalence of Maintenance Care Consultation patterns
Malmqvist 2009 [12]	Structured workshop, a focus group discussion	Chiropractors	*N* = 15	NA	Indications for Maintenance Care
Sandnes 2010 [13]	Observation in clinics	Chiropractors	*N* = 868	NA	Prevalence of Maintenance Care Consultation patterns Decision making
Hansen 2010 [15]	Survey	Chiropractors	*N* = 297	72%	Indications for Maintenance Care Prevalence of Maintenance Care Chiropractor-related factors associated with Maintenance Care
Senna 2011 [18]	RCT	Patients from specialized hospital clinic	*N* = 60	65%	Efficacy of intense follow up with SMT compared to a) SMT without follow up and b) sham SMT without follow up
Martel 2011 [19]	RCT	Chiropractic Patients	*N* = 98	93%	Efficacy of SMT compared to a) SMT plus exercise and b) attention
Cifuentes 2011 [21]	Observational, database	Patients with workers’ compensation claims	*N* = 894	NA, selected group	Health care use for chiropractic patients compared to physician- and physical therapist- patients
Bringsli 2012 [10]	Observational + Survey	Chiropractors + Chiropractic Maintenance Care-patients	*N* = 178 *N* = 373	NA Not known	Rationale Consultation patterns Content
Axén 2013 [9]	Observational	Chiropractors	*N* = 252	96%	Indications for Maintenance Care
Myburgh 2013 [14]	Interview study	Chiropractors	*N* = 10	NA	Definition of concept Indications for Maintenance Care Consultation patterns Content Decision making
Eklund 2018 [16]	RCT	Chiropractic patients	*N* = 319	97%	Efficacy of pre-scheduled treatments compared to treatments when needed.
Maiers 2018 [17]	RCT	Chiropractic patients	*N* = 180	90%	Efficacy of SMT and exercises for 36 weeks compared to SMT and exercises for 12 weeks.

*SMT* Spinal Manipulative Therapy, NA Not applicable, *RCT* Randomized controlled trial

### The concepts of maintenance care

#### Rationale

Some studies explored the concept of Maintenance Care in terms of its rationale, as defined by chiropractors [11, 14, 20]. They clearly described Maintenance Care as a *type of prolonged care delivered at regular intervals.*

In interviews with chiropractors, Maintenance

Care was described as a *preventive* approach, aimed at preventing new episodes and maintaining improvement, i.e. secondary and tertiary prevention [11, 14].

One study specifically investigated the patient perspective of Maintenance Care [20]. In this study, Maintenance Care patients were interviewed and asked to explain, why they would visit their chiropractor on a regular basis. Their answers were in accordance with those of the chiropractors, i.e. patients stated that the purpose was to prevent recurrences (78%) and to remain pain-free (68%). A few patients (17%) echoed the holistic view expressed by some chiropractors, i.e. that Maintenance Care was used to prevent disease in general, a wellness approach.

#### Spacing of maintenance care treatments

Four studies explored the pattern of consultation for Maintenance Care patients, two through interviews [11, 14] and two by extracting consultation dates from patient files. Both interview studies found 3 months intervals to be the most common [11, 14]. More exact information was collected from patient files, which showed a mean of 9 weeks and a mode of 3 months between visits [20] and that most visits were scheduled within a range of one to 3 months [13].

#### Content of maintenance care

An interview study of Danish chiropractors showed that Maintenance Care sessions included a range of treatment modalities, from the ordinary examination/manual treatment to packages including exercise prescriptions, advice on ergonomics, diet, weight loss, and stress management, i.e. it included a program meant to motivate patients to maintain healthy lifestyle habits through empowerment [14]. The Maintenance Care session was described as a “check-up”, where also public health issues, such as exercise and healthy living, were addressed. However, the role of strength- and conditioning- training, particularly, appeared to be a cause of disagreement between the interviewed chiropractors.

The interview findings were not reproduced in a study in which both Maintenance Care and non- Maintenance Care consultations were observed, as very little difference was found between the two in terms of duration and content. Further, Maintenance Care patients were observed to be treated in more areas of the spine and more often with a “full” spine perspective compared to the non- Maintenance Care patients [20], which had not been evoked in the interview study.

### The indications for the use of maintenance care

The indications for the use of Maintenance Care had been studied in different ways, from focus groups [10, 12] to surveys [8, 10, 15]. Only the most common findings are aggregated here. Studies, including specific hypothetical cases, found that Maintenance Care was offered, particularly, if the patient had had *previous episodes* and had *improved* with treatment [8, 15]. When clinicians were asked to identify suitable cases, the patient’s past history was also acknowledged [12]. These indications were tested in an observational study where chiropractors were asked to judge if their patients were suitable for Maintenance Care or not. Patients’ baseline data were then used as predictors in a regression analysis with this judgement as outcome, and previous episodes were, indeed, found to be the strongest predictor for recommending Maintenance Care [9].

However, clinicians discussing/ being interviewed also stated that patient-related factors such as being worried, having a stressful life or a hard physical job, would be considered before recommending Maintenance Care [10, 14], and chiropractors participating in a work-shop on this topic also mentioned the patient-doctor-relationship as an important point to consider before recommending Maintenance Care [12].

Some chiropractors deviated from the mainstream and mentioned that “a perception of sub-optimal biomechanical function” would be an indicator for recommending Maintenance Care [14]. Thus, a small group of chiropractors seemed more oriented towards their own clinical findings rather than the patients’ symptoms.

### Chiropractors’ belief in maintenance care

Upon a direct question in a survey, the majority of chiropractors (98%) stated that they believed that Maintenance Care could be used as a preventive tool, at least sometimes [10]. Probing this topic further, a study of various LBP scenarios showed that there were some clinicians, who chose Maintenance Care above that of prevention of recurrent and persistent pain [8]. This was further highlighted in an interview study, where it was found that some chiropractors favored a universal approach, claiming that Maintenance Care was always beneficial and would prevent disease [14]. This approach may be considered a primary preventive approach, commonly called “wellness”, again, focusing on findings rather than symptoms and past history.

### Patients’ acceptance of maintenance care

We found no studies that examined to what extent patients accept the concept of Maintenance Care or the proportion of patients, who took up the offer of Maintenance Care (e.g. by calculating the proportion of patients who accepted an offer of Maintenance Care out of the patients who were given this offer).

In an RCT of Chiropractic Maintenance Care, patients’ expectations of improvement were investigated. Positive expectations were found to decrease during the duration of the study, more so among patients treated for a short time compared to patients with a longer follow up [17].

### The prevalence with which chiropractors use maintenance care

Some studies investigated the frequency of use of Maintenance Care from simply asking chiropractors to estimate their use of Maintenance Care the previous week (mean estimate 22%) [15], to have them check the proportion on a typical clinic day (reported in two studies to be 28 and 35%, respectively) [8, 11], or actually observing in clinic and counting (reported in two studies to be 26 and 41%, respectively) [13, 20]. Thus, the mean proportion of patents seen on a Maintenance Care regimen by Scandinavian chiropractors was around 22–41%, with large individual variations ranging from 0 to 100%.

### Chiropractor characteristics associated with maintenance care use

One study conducted in Denmark investigated chiropractic factors associated with Maintenance Care use and found that it was more common among experienced chiropractors, clinic owners, and those who received their chiropractic degree in the US (as opposed to colleagues trained in Europe) [15]. However, at the time of the study, the older chiropractors were almost all trained in the US, whereas the younger chiropractors were primarily educated in Denmark. Therefore, it is not known, if it is age (experience) or educational background that guided the use of Maintenance Care among these chiropractors.

### The clinical usefulness of maintenance care

The two previous reviews of studies on Maintenance Care failed to reveal any studies on clinical usefulness. The Nordic Maintenance Care program was initiated to make it possible to identify the indications for Maintenance Care to make such studies possible. In this new review, we found four RCTs that investigated the clinical outcome of repeated treatments over a prolonged period. These trials are summarized in Table 2. Two studies investigated the outcome on patients with LBP [16, 18], one studied NP [19], and one study included patients with both LBP and NP [17].

**Table 2:** A summary of the included RCTs and their use of evidence regarding indications for Maintenance Care and frequency of care, as well as the outcomes and when they were measured

Author, year of publi-cation	Sample size, Number of clinicians	Inclusion criteria:			Contents and frequency of Maintenance Care visits	Comparator(s)	Time of follow-ups	Outcome - = no effect + = effect
		Previous episodes	Persistent pain	Improvement with initial care				
Senna 2011 [18]	*N* = 60 3 MDs	–	x	–	A month of initial care followed by Maintenance Care every 2 weeks for 9 months	1: Sham SMT for a month without continued care 2: SMT for a month without continued care	After 1, 4, 7 and 10 months	Disability + Pain + Health +
Martel 2011 [19]	*N* = 98 3 DCs	–	x	–	Active Treatment: Monthly & Advice: every 2nd month for 10 months	1: SMT + Exercise 2: Attention only	After initial treatment, mid-trial and end of trial	Pain – Function – Disability +
Maiers 2018 [17]	*N* = 180 1 clinic with DCs + ETs	–	x	–	Monthly for 36 weeks	1: Treatment for 12 weeks	Weeks 4, 12, 34, 36, 52 and 78.	Disability -
Eklund 2018 [16]	*N* = 319 35 DC’s	X	x	X	Every 1–3 months for 12 months	1: Treatment after symptoms reoccur on patient’s initiative	Weekly for 52 weeks	Number of days with bothersome pain +

*DC* Doctor of Chiropractic, *ET* Exercise Therapist, *SMT* Spinal Manipulative Therapy

One study compared groups who received either Maintenance Care or self-managed appointments [16], and three studies compared groups with different content and treatment duration [17–19]. However, only one of these studies [14] used the inclusion criteria for Maintenance Care that were identified through the Nordic Maintenance Care Program. These criteria were: recurring problems that improved well with initial treatments.

Three of the RCTs specifically stated that they were dealing with chiropractic Maintenance Care [16, 17, 19], whereas one was set in a secondary care setting, with medical doctors as therapists treating chronic LBP [18]. Comparison in that study were made between i) manual treatment with an intense program of continued care over 9 months and ii) short term manual treatment without continued care and iii) sham manual treatment without continued care. The results favored the group receiving manual treatment and long-term intense continued care.

In the oldest chiropractic study, patients with chronic NP were randomized after an initial course of treatment to receive Maintenance Care with different content: Spinal Manipulative Therapy (SMT) or SMT plus exercise [19]. These treatment groups were compared to a group of patients who received attention only, but no differences were observed in pain or function between the groups after 10 months.

Both of these studies [18, 19], may suffer from a “proximity effect of treatment to follow-up”, as outcomes were measured close to the last treatment and may not reflect the outcome over the full follow-up period, and outcomes may therefore be inaccurate.

In a later study, elderly patients with NP and LBP all received Maintenance Care in the form of SMT and supervised exercises [17]. Patients were randomized to different durations of treatment, but patients treated for 36 weeks showed no significant improvements in disability compared to patients treated for 12 weeks.

In the fourth study, Eklund et al. made use of the accumulated evidence regarding indications, treatment approach and spacing of treatments for Maintenance Care. Based on the profile of the patient presumed suitable for Maintenance Care, patients with recurrent (episodic) or persistent LBP, who improved on an initial course of chiropractic treatment (i.e. definitely improved by the 4th visit) were included in a multicenter study [16]. The participating chiropractors had been selected because they thought Maintenance Care could be useful but did not use it on all their patients. A significant difference with 13 days less with bothersome pain over 12 months was noted in the group randomized to follow-up sessions scheduled at regular intervals, compared to patients who were told to come back only when their symptoms recurred. The mean additional number of treatments with the Maintenance Care treatment was 2 visits over 1 year.

### Cost-effectiveness of maintenance care

We found no studies of cost-effectiveness of Maintenance Care. However, a health-care register-based study found that health care use was smallest among the patients who received Maintenance Care from a chiropractor as opposed to those receiving care from other health care professionals [21]. However, the patient groups were not randomly allocated to one type of treatment or another and were not compared for similarities and differences at base line, making it difficult to know if such an analysis was meaningful.

## Discussion

### Brief summary of findings

This narratively reported systematic review, the third over 20 years, has been able to describe more fully the concept of Maintenance Care and to report on its clinical usefulness.

### The concept of maintenance care

It is now clear that Maintenance Care is mainly seen as a secondary or tertiary preventive approach, used in various ways. The application of Maintenance Care varies, from clinicians who never provide any Maintenance Care to those who suggest that all patients should be put on an Maintenance Care scheme. Across the Nordic countries, around 30% of chiropractic patients are Maintenance Care patients. *When* it is provided, visits are usually scheduled between 1 and 3 months. Interestingly, what chiropractors believe that they do and what they actually do during these sessions, do not necessarily concur. The intent is to have a public health approach, but observations indicate that Maintenance Care sessions resemble ordinary consultations although with an emphasis on a full spine approach. The clinical indications vary, but patients suitable for Maintenance Care are commonly thought to be those with persistent or episodic pain, who react well to the initial treatment.

### The clinical usefulness of maintenance care

Three trials dealt with the clinical usefulness of Maintenance Care, without considering the indications for treatment unearthed through the Nordic Maintenance Care Program. In one, patients who received Maintenance Care had better outcome than those who received short-term treatment or short-term sham treatment [18]. The other two studies compared two types of Maintenance Care (with or without exercises, or different length of the follow-up treatments) and found no difference of outcomes between groups [17, 19].

The multicenter trial that was designed specifically on the basis of the results from the 10 year long research program found a considerable difference in the number of bothersome days, favoring the Maintenance Care group to the one which was encouraged to ‘call when needed’ [16].

### Clinical considerations

Maintenance Care can clearly be said to be used as a preventive therapeutic concept, although the exact interpretation varies somewhat between chiropractors. The logical approach would obviously be to provide this type of treatment on patients who initially get better with chiropractic care and to do so for as long as it seems useful from the patient’s perspective. However, trajectories vary between patients but also within subjects, with symptoms changing over time. It is therefore important to be vigilant regarding new developments and reassessments of patients’ symptoms. Chiropractors could obviously play an important role here as ‘back pain coaches’, as the long-term relationship would ensure knowledge of the patient and trust towards the chiropractor. This should ideally result in an individualized treatment approach to improve the long-term trajectories.

### Methodological considerations

We conducted a systematic search for this review, but did not consider methodological quality of the reviewed studies, as they consisted of many different research designs and were able to illuminate the Maintenance Care concept from several angles. Qualitative studies were used to explore clinicians’ and patients’ views on Maintenance Care in detail, and surveys based on these results were used to consolidate these findings. Observations in clinics were able to describe the Maintenance Care treatment content, and observational studies to consolidate the findings regarding indications. Finally, RCTs examined the usefulness of the Maintenance Care approach. The findings reported here are not necessarily an accurate picture of the use and usefulness of Maintenance Care. However, they are logical and the indications for treatment were used in the ensuing clinical trial that showed the best results. Therefore, chiropractors who use the inclusion criteria of episodes and improvement have perhaps, through experience or intuition, identified the best method of approach.

Most of the studies originated in Northern Europe, for which reasons these findings may not be transferable to other countries and other cultures and probably differ according to the ‘origin’ of the chiropractor.

### Perspectives and conclusions

Back pain is a chronic disease for most, with episodes at short or long intervals. A preventive approach such as Maintenance Care, therefore, makes sense. It is still not known if it ‘works’ because of the treatment given or because of the clinical encounter, or how these two components interact. Further studies are therefore needed both to investigate the patients who respond best to Maintenance Care treatment and which components of the treatment session that are most valuable and for whom.

## Supplementary information

**Additional file 1.** Search strategy.

## Data Availability

Data sharing is not applicable to this article as no datasets were generated or analyzed during the current study.
