# Correction to: Chiropractic maintenance care - what’s new? A systematic review of the literature

**DOI:** 10.1186/s12998-020-0301-8

**Published:** 2020-01-28

**Authors:** Iben Axén, Lise Hestbaek, Charlotte Leboeuf-Yde

**Affiliations:** 1 Karolinska Institutet, Institute of Environmental Medicine, Unit of Intervention and Implementation Research for Worker Health, Nobels väg 13, 171 77 Stockholm, Sweden; 2 Et Liv I Bevegelse (The Norwegian Chiropractic Research Foundation), Storgt 10a, 0155 Oslo, Norway; 3 Deptartment of Sport Science and Clinical Biomechanics, Odense, Denmark; 4 Nordic Institute of Chiropractic and Clinical Biomechanics, Odense, Denmark; 5 Institute for Regional Health Research, University of Southern Denmark, Odense, Denmark

**Correction to: Chiropr Man Therap (2019) 27:63**

**https://doi.org/10.1186/s12998-019-0283-6**

After publication of our article [1] the authors have notified us that their names have been incorrectly tagged.

- Original name tagging:

Axén Iben

Hestbaek Lise

Leboeuf-Yde Charlotte

- Correct name tagging:

Iben Axén

Lise Hestbaek

Charlotte Leboeuf-Yde

The original article has been corrected.
