# Supplementary material for: Chiropractic maintenance care - what’s new? A systematic review of the literature
Source: Chiropr Man Therap. 2019 Nov 21;27:63. doi: 10.1186/s12998-019-0283-6 (PMC6868774; doi:10.1186/s12998-019-0283-6)
Supplement: Supplementary file 1 — Additional file 1. Search strategy. [file 12998_2019_283_MOESM1_ESM.pdf]

Additional File 1: Search strategy

(("chiropractic"[MeSH Terms] OR "chiropractic"[All Fields]) AND ("musculoskeletal manipulations"[MeSH Terms] OR ("musculoskeletal"[All Fields] AND "manipulations"[All Fields]) OR "musculoskeletal manipulations"[All Fields] OR ("manual"[All Fields] AND "therapy"[All Fields]) OR "manual therapy"[All Fields])) AND (("long-term care"[MeSH Terms] OR ("long-term"[All Fields] AND "care"[All Fields]) OR "long-term care"[All Fields] OR ("maintenance"[All Fields] AND "care"[All Fields]) OR "maintenance care"[All Fields]) AND ("prevention and control"[Subheading] OR ("prevention"[All Fields] AND "control"[All Fields]) OR "prevention and control"[All Fields] OR "prevention"[All Fields]))
